# Evaluation of free and total fluoride concentration in mouthwashes via measurement with ion-selective electrode

**DOI:** 10.1186/s12903-019-0908-0

**Published:** 2019-11-20

**Authors:** Vladimir Yu. Reshetnyak, Olga V. Nesterova, Oleg I. Admakin, Denis A. Dobrokhotov, Irina N. Avertseva, Samira A. Dostdar, Dinara F. Khakimova

**Affiliations:** 1 Department of Chemistry in the Institute of Pharmacy, Sechenov University, Moscow, Russia; 2 Department of Prevention and Communal Dentistry, Institute of Dentistry, Sechenov University, Moscow, Russia; 3 5th year undergraduate student of the Institute of Pharmacy, Sechenov University, Moscow, Russia

**Keywords:** Fluorides, Mouthwashes, Potentiometry, Ion-selective electrode

## Abstract

**Background:**

The aim of this study was to compare free fluoride concentration and total fluoride concentration in mouthwashes.

**Methods:**

Fluorine-containing mouthwashes from various companies and manufacturers (Colgate Total Plax Classic Mint®, Colgate-Palmolive, New York, USA; Colgate Total Plax Gentle Mint®, Colgate-Palmolive, New York, USA; Colgate Total Plax Fresh Mint®, Colgate-Palmolive, New York, USA; Oral B Advantage®, Procter&Gamble, Cincinnati, USA; Reach Fresh Mint®, Johnson&Johnson, New Brunswick, USA; Foramen®, Laboratorios Foramen, Guarnizo, Spain; Lacalut Sensitive®, Dr. THEISS, Homburg, Germany; Sensodyne®, GlaxoSmithKline, London, UK; Vesna F®, Vita, Saint Petersburg, Russia; Lacalut Fresh®, Dr. THEISS, Homburg, Germany) were selected as study objects. Fluoride measurements were carried out using the fluoride selective electrode.

**Results:**

Free fluoride:total fluoride ratio was more than 80% for six samples (Colgate Total Plax Gentle Mint® - 88%, Colgate Total Plax Fresh Mint® - 99%, Oral B Advantage® - 92%, Reach Fresh Mint® - 92 and 89% for the mouthwash of another batch, Lacalut Sensitive® - 94%) and less than 63% for three samples (Colgate Total Plax Classic Mint® - 56%, Foramen® - 62%, Vesna F® - 61%). Two samples had more than 70% and less than 80% of unbound fluoride, respectively (Sensodyne® - 77%, another batch of Oral B Advantage® mouthwash - 74%). Rinse containing sodium monofluorophosphate (Na_2_PO_3_F) (Vesna F®) had more than 50% of free fluoride, while the rinse containing amine fluoride (AmF) (Lacalut Sensitive®) had 94%. The difference in the free fluoride:total fluoride ratio can be explained by binding of fluoride ions by components contained in mouthwashes, such as coloring agents and polymeric compounds. The lowest concentration of free fluoride ions (0.000093 mol/L) was observed for aluminum fluoride (AlF_3_) rinse (Lacalut Fresh®), while the total fluoride amount was not determined due to possible generation of strong fluoride complexes. This implies that fluoride ions will not be uptaken by tooth tissue and may even be washed away from it, compromising the efficacy of mouthwashes.

**Conclusions:**

The differences in free fluoride: total fluoride ratio between analyzed mouthwashes reveal a need to develop a method for evaluation of free fluorides in mouthwashes for proper updating of national and international guidelines.

## Background

According to the World Health Organization, dental caries is a disease prevalent among 60–90% of school-age children and almost 100% of adults worldwide [1]. There are numerous reports regarding the influence of comprehensive prevention programs on the prevalence of caries, including topical fluoridation provided by the application of fluoride-containing toothpastes, mouthwashes, gels, foams, and varnishes [2–8].

In addition to the fluorides, other inorganic elements are essential to mineralize hard tooth tissues and increase their resistance to caries. Toothpastes and mouthwashes containing sodium phosphate, calcium glycerophosphate, and zinc oxide exhibit a pronounced anticaries action [9–11].

Mouthwashes are additional products for oral hygiene. Most of the currently available mouthwashes can be divided into four groups, depending on their therapeutic action: (1) mouthwashes reducing oral malodor; (2) mouthwashes decreasing dental plaque formation due to antibacterial action; (3) mouthwashes containing various concentrations of fluorine compounds and able to affect the mineralization of dental hard tissue; and (4) mouthwashes preventing or reducing gingivitis [12–17].

Various fluorine and phosphorus compounds are included as active components in the mouthwashes of the second and the third groups. Basic organic and inorganic fluorine compounds, contained in products for prophylaxis of oral cavity diseases, are sodium fluoride (NaF), sodium monofluorophosphate (Na_2_PO_3_F), amine fluoride (AmF), aluminum fluoride (AlF_3_), and stannous fluoride (SnF_2_) [18, 19]. The active mineral supplements could be phosphate salts, for instance, nano-sized sodium hexametaphosphate and sodium trimetaphosphate, which are capable of changing the solubility of enamel after adsorption on its surface [20, 21].

Mouthwashes containing fluorine compounds are divided into groups, depending on fluoride ion concentration: 0.05% sodium fluoride solutions can be used daily; and 0.2% sodium fluoride solutions are recommended for weekly or fortnightly application [22].

Rošin-Grget et al. reviewed various theories of the cariostatic action of fluorine. According to one of these theories, fluoride ions penetrate into the lattice of hydroxyapatite, Ca_10_(PO_4_)_6_(OH)_2_, resulting in the formation of fluorohydroxyapatite, Ca_10_(PO_4_)_6_(OH) F, which is more resistant to acids [23].

According to another theory, during the exposure of the tooth surface to NaF, the reaction with hydroxyapatite results in the formation of poorly soluble crystals of calcium fluoride [23]:

$$ {\mathrm{Ca}}_{10}{\left({\mathrm{PO}}_4\right)}_6{\left(\mathrm{OH}\right)}_2+20\mathrm{NaF}\to 10{\mathrm{Ca}\mathrm{F}}_2\downarrow +{6\mathrm{Na}}_3{\mathrm{PO}}_4+2\mathrm{NaOH} $$

It should be noted that the equilibrium shift in both chemical processes is determined by the activity of fluoride ions in the oral fluid, which in turn depends on the concentration of free fluoride ions in the applied prophylactic and therapeutic preparations. Various ingredients of mouthwashes, such as colourants, flavouring agents, sweeteners, preservatives, and surfactants, can chemically bind fluoride ions. Such bound fluoride is not effective in preventing and reducing dental caries. Thus, the control and the determination of «active» fluoride can determine the quality and, hence, the therapeutic efficacy of mouthwashes.

Fluorine-containing mouthwashes currently used in dental practice can be divided into three types: (1) therapeutic mouthwashes that can be purchased over-the-counter or prescribed only under the supervision of a doctor; (2) cosmetic mouthwashes mainly aimed at freshening breath; and (3) mouthwashes that are a combination of these two types [24].

Since the first type consists of therapeutic agents, the content of biologically active compounds is controlled by the corresponding normative and technical documentation and by appropriate methods of quantitative analysis of total fluoride.

The quality of the second type of preparations is in many cases determined only by the technical specifications in manufacturing and may not provide for the quantitative determination of the active substances in the final product.

The aim of this study was to compare active-fluoride and total-fluoride concentrations in 10 mouthwashes commercially available in Russian Federation.

## Methods

### Fluoride products

Fluorine-containing mouthwashes were purchased from local markets of Moscow (Russian Federation) and selected as study objects (Table 1). Colgate Total Plax Classic Mint® (0.025% NaF), Colgate Total Plax Gentle Mint® (0.025% NaF), and Colgate Total Plax Fresh Mint® (0.025% NaF) are produced by Colgate-Palmolive (New York, USA). Each of the products exhibits antibacterial activity and prevents dental plaque formation and oral malodor. Procter&Gamble (Cincinnati, USA) produces Oral B Advantage® (0.05% NaF) mouthwashes aimed at providing tooth and gum care. The company producing Reach Fresh Mint® is Johnson&Johnson (New Brunswick, USA). In addition to NaF (the content was not declared), Reach Fresh Mint® contains another active component – cetylpyridinium chloride. Laboratorios Foramen (Guarnizo, Spain) produces Foramen® (0.05% NaF), which strengthens tooth enamel. Dr. THEISS (Homburg, Germany) produces Lacalut Sensitive® (0.33% AmF) and Lacalut Fresh® (0.2% AlF_3_). Lacalut Sensitive® decreases sensitivity of teeth to thermal stimuli. Lacalut Fresh® prevents parodontitis due to astringent properties of aluminum lactate. GlaxoSmithKline (London, UK) is manufacturer of Sensodyne® (0.0217% NaF). Sensodyne® also reduces tooth sensitivity. Vita (Saint Petersburg, Russia) produces Vesna F® (0.012% fluorine) mouthwash, which contains Na_2_PO_3_F and calcium glycerophosphate as active components. These mouthwashes are the most widely represented on Russian market and recommended by dental practitioners for the proper oral care.

**Table 1:** Composition of fluoride-containing mouthwashes.

Brand	Composition					
	Active ingredients		Et	Glyc	Sorb	Other ingredients
	Fluorides	Phosphates				
Colgate Total Plax Classic mint®	NaF	Disodium hydrogen phosphate	+	+	+	Sodium lauryl sulfate, sodium methyl cocoyl taurate, menthol, saccharin, aqua, flavourings, colourants
Colgate Total Plax Gentle mint®	NaF	Disodium hydrogen phosphate	+	+	+	Sodium lauryl sulfate, sodium methyl cocoyl taurate, menthol, saccharin, aqua, flavourings, colourants
Colgate Total Plax Fresh mint®	NaF	Disodium hydrogen phosphate	+	+	+	Sodium lauryl sulfate, sodium methyl cocoyl taurate, menthol, saccharin, aqua, flavouring agents, colourants
Oral B Advantage®	NaF	–	+	+	–	Methylparaben, propylparaben, poloxamer 407, cetylpyridine chloride, sodium saccharin, aqua, flavouring agents, colourants
Reach Fresh mint®	NaF	Disodium hydrogen phosphate, sodium dihydrogen phosphate	+	+	+	copolymer РОЕ, sodium benzoate, polyphenylene oxide, POE (20) sorbitan monooleate, perfume, cetylpyridinium chloride, aqua, colourants
Foramen®	NaF	–	–	+	+	Potassium nitrate, PEG-40, allantoin, flavour additive, sodium methylparaben, sodium propylparaben, lemon acid, sodium benzoate, cetylpyridine chloride, aqua, sodium saccharinate
Lacalut sensitive®	AmF	–	–	+	–	Cocamidopropyl betaine, chlorhexidine bigluconate, aqua, aluminum lactate, propylene glycol, PEG-40, flavouring agent
Sensodyne®	NaF	Disodium hydrogen phosphate, sodium dihydrogen phosphate	–	+	+	Sodium benzoate, poloxamer 338, potassium chloride, PEG-60, aqua, cetylpyridine chloride, sodium saccharinate, sodium chloride, methylparaben, flavoring agent
Vesna F®	Na_2_PO_3_F	_	_	_	_	Aqua, xylitol, calcium glycerophosphate, polyoxyethylene sorbitan monooleate, sodium benzoate, citric acid, food flavour, colourants
Lacalut Fresh®	AlF_3_	_	_	_	_	Aqua, isopropyl alcohol, PEG-40 hydrogenated castor oil, flavors, peppermint extract, aluminum lactate, chlorhexidine bigluconate, sodium saccharinate, allantoin, bisabolol

*Et* ethanol, *Glyc* glycerol, *Sorb* sorbitol

### Fluoride measurements

The method of direct potentiometry was used to determine fluoride ion activity using fluoride ion–selective electrode based on lanthanum fluoride. Standard silver chloride electrode was used as a reference electrode. Electromotive force of a galvanic cell was measured by standard laboratory ion meter И160М. The calibration of the electrode was performed every day before fluoride measurements.

**Fig. 1:**
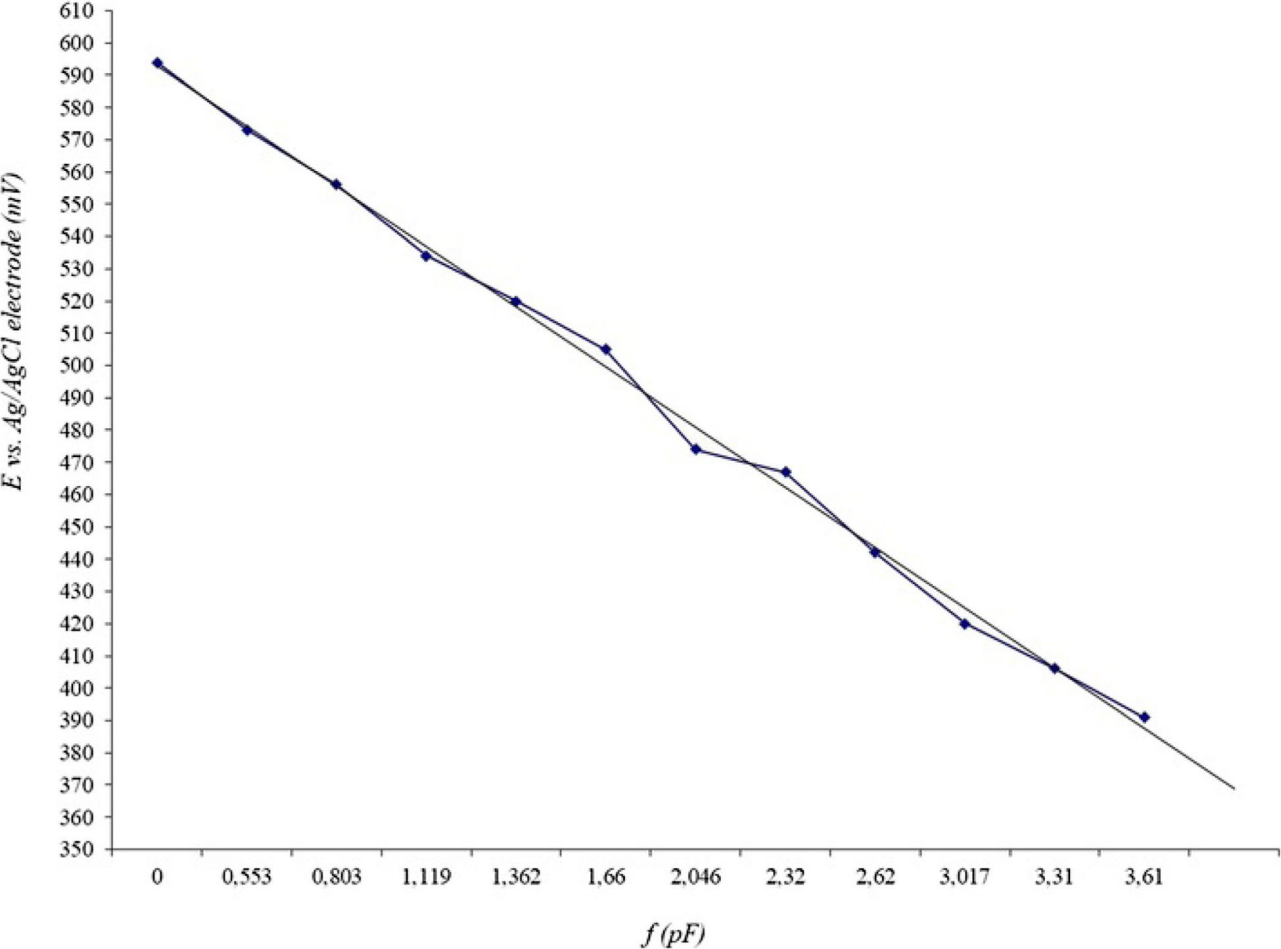
Calibration curve representing electrode response to fluoride concentration at T = 298 K

To obtain calibration curves, series of NaF standard solutions (5·10^− 1^-5·10^− 5^ M) were prepared using either deionized water, or Albavit solution, which does not contain fluoride ions and is similar in composition to the test mouthwashes. Calibration curve data is given in Table 2 and Table 3 and in Fig. 1. The accuracy of measurement of fluoride ion concentrations in standard solutions of NaF is shown in Table 4. Similar measurements for NaF solution prepared with Albavit solution are represented in Table 5.

**Table 2:** Calibration of ISE in NaF standard aqueous solutions

	Concentration of NaF, mol/L									Average
	0.5	0.1	0.05	0.01	0.005	0.001	0.0005	0.0001	0.00005	
рF	0.533	1.119	1.362	2.046	2.320	3.017	3.310	4.000	4.301	
EMF	573.0	532.0	524.2	470.0	466.9	409.0	405.5	353.0	338.0	
	573.7	531.5	524.7	471.0	467.6	407.6	406.6	350.0	344.4	
	572.8	532.3	522.0	471.0	469.2	406.7	408.0	347.6	340.0	
		534.4		478.0		410.0		340.0		
		536.0		478.0		407.0		345.5		
		539.5		473.8		404.0		348.1		
X̅	573.2	534.3	523.6	473.6	467.9	407.4	406.7	347.4	340.8	
S	0.47	3.03	1.44	3.61	1.18	2.08	1.25	4.40	3.27	2.18
Δ X	0.69	3.18	2.14	3.79	1.75	2.18	1.86	4.62	4.86	2.53
E(%)	0.12	0.60	0.41	0.80	0.37	0.53	0.46	1.33	1.43	0.58

**Table 3:** Calibration of ISE in NaF solutions prepared with Albavit solution

pF	0	0.553	0.803	1.119	1.362	1.660	2.046	2.320	2.620	3.017	3.310
EMF (aq.)	594	573		534	520		474	467		420	406
EMF (Alba)			556			505			442

**Table 4:** The accuracy of measurement of F^−^ in NaF standard aqueous solutions

NaF / TISAB	EMF	+ 10 ml TISAB	V (NaF) added, ml	ΔЕ	с (F^−^)	%	X̅	S	X	E, %
5 / 5	508.2	490.9	9.2	17.3	0.096	96	95.8	1.92	2.21	2.3
3 / 7	495.6	478.5	4.0	17.1	0.095	95				
7 / 3	518.4	498.7	19.2	19.7	0.094	94				
3 / 7	497.0	478.8	4.2	18.2	0.099	99				
5 / 5	505.0	488.0	9.1	17.0	0.095	95

**Table 5:** The accuracy of measurement of F^−^ in NaF standard solutions prepared using Albavit solution (4 ml «Alba» + 1 ml NaF)

c (NaF), mol/L	EMF	+ 5 ml TISAB	+ 10 ml TISAB	V (NaF) added, ml	с (F^−^) theoretical	с (F^−^) actual	c̄ (F^−^)
0.5	532.2	505.3	486.9	1.0	0.100	0.104	0.105 ± 0.002
0.5	536.7	509.7	490.8	1.0	0.100	0.106	
0.5	534.2	507.2	489.8	1.0	0.100	0.105	
0.1	501.3	469.5	450.8	1.0	0.020	0.022	0.022 ± 0.0005

To determine total fluoride concentration in mouthwashes, the method of standard addition was applied. Samples of mouthwashes were diluted in total ionic strength adjustment buffer (TISAB), which sets constant ionic strength during measurement and which also precomplexes interfering ions (e.g., aluminium ions Al^3+^). After the measurement of potential of each diluted sample a known amount of standard was added and the measurement was repeated again for further calculation of difference in the potentials and quantification of total fluoride. Since the results of quantitative determination of fluoride ions on standard mixtures prepared with aqueous solution and Albavit solution were slightly differentiated (i.e., the results obtained in the first case were systematically lower compared to those obtained theoretically, whereas in the second case the results were found to be higher), we attempted to modify the procedure by using the method of double increase in concentration without preliminary dilution with TISAB solution in order to determine possible binding of fluoride ions by components contained in the mouthwashes. Every sample of the mouthwash was measured five times and the mean of the measurements was used for further statistical estimation.

## Results

Results of fluoride analysis are shown in Table 6. Most of the analyzed mouthwashes contained NaF as a source of fluoride ions; other types of fluoride agents were Na_2_PO_3_F, AmF, and AlF_3._ Free fluoride:total fluoride ratio was more than 80% for six samples (Colgate Total Plax Gentle Mint® - 88%, Colgate Total Plax Fresh Mint® - 99%, Oral B Advantage® - 92%, Reach Fresh Mint® - 92 and 89% for the mouthwash of another batch, Lacalut Sensitive® - 94%) and less than 63% for three samples (Colgate Total Plax Classic Mint® - 56%, Foramen® - 62%, Vesna F® - 61%). Two samples had more than 70% and less than 80% of unbound fluoride, respectively (Sensodyne® - 77%, another batch of Oral B Advantage® mouthwash - 74%). Rinse containing Na_2_PO_3_F (Vesna F®) had more than 50% of free fluoride, while the rinse containing AmF (Lacalut Sensitive®) had 94%. The lowest concentration of free fluoride ions (0.000093 mol/L) was observed for AlF_3_ rinse (Lacalut Fresh®).

**Table 6:** The determination of fluoride-ions concentration and activity by the method of standard addition and modified method

Label	pH	Dilution	F^−^, mol/L × 10^−3^			
			Method of standard addition		Modified method	
			c (F^−^)	a (F^−^)	c (F^−^)	a (F^−^)
Colgate Total Plax Classic mint	7.15	–	9.77	5.52	9.92	5.43
		1÷10	0.75	0.89	1.11	1.06
Colgate Total Plax Gentle mint	5.70	–	9.14	8.05	8.91	8.71
		1÷10	1.22	0.75	1.19	1.20
Colgate Total Plax Fresh mint	5.95	–	9.87	9.81	9.39	8.92
		1÷10	0.63	0.47	0.70	0.43
Oral B Advantage	5.70	–	17.23	15.81	25.91	15.52
		1÷10	1.42	1.21	1.2	0.98
Oral B Advantage^a^	5.75	–	16.70	12.30	16.81	6.08
Reach Fresh mint	5.80	–	15.86	14.56	24.09	13.71
		1÷10	1.20	0.71	1.40	1.02
Reach Fresh mint^a^	5.75	–	18.15	16.10	26.90	14.20
Foramen	5.80	–	14.62	9.11	20.05	9.05
		1÷10	0.75	0.94	1.20	1.07
Lacalut sensitive	4.40	–	5.48	5.13	8.60	4.20
		1÷10	0.758	0.77	0.15	0.86
Sensodyne	5.70	–	16.25	12.53	23.59	11.12
		1÷10	2.01	1.27	–	–
Vesna F	6.05	–	4.49	2.76	5.20	2.71
Lacalut Fresh	4.20	–	–	0.093	1.30	0.095
		1÷10	–	0.012	0.14	0.012

^a^ mouthwash of another batch was used

## Discussion

The State Standard of Russian Federation Р 51577–2000 specifies only the total amount of fluoride ions in liquid oral hygiene products. We failed to find articles dedicated to the determination of free fluoride concentration in mouthwashes; however, several authorities have been focusing on the studies aimed at free fluoride analysis in toothpaste since 2005 [25–27]. The participants of European Organization for Caries Research Workshop, which was held to discuss the issues related to the methodology for determination of potentially available fluoride in toothpastes, have emphasized the necessity of developing methods measuring fluoride bioavailability [28]. The development of techniques providing the assessment of free fluoride in mouthwashes is also required. Mouthwashes selected for fluoride measurements do not represent all the variety of brands commercially available in Russia, however even the small sample size indicates great discrepancies between the obtained data on fluoride content.

The experiment showed the results to be almost identical for the Colgate Total Plax (Gentle Mint)® and Colgate Total Plax (Fresh Mint)® solutions, with both methods.

Significant differences in concentrations determined by the method of standard addition and by the modified method of quantitative assessment were observed for such solutions as Oral B®, Reach®, and Foramen®. The fact that an increase in total fluoride content was observed with the modified method may be explained by a change in the ionic strength of the solution during the measurement; or by partial binding of fluoride ions by certain components of the mouthwashes; or possibly by polymeric compounds such as poloxamers and polyphenylene oxides. Analogous observations were made for the Sensodyne® solution, which does include polymeric compounds. This assumption is supported by the fact that in standard solutions prepared with Albavit solution containing polymeric compounds, the determined concentration was systematically higher than the theoretical concentration; whereas systematically lower results were obtained with model solutions in the procedure of fluoride ion determination using TISAB solution.

In those preparations lacking polymeric compounds, results of fluoride ion determination were identical at the constant ionic strength and modified method within the error of experiment. To confirm these assumptions, we determined concentration and activity of the test solutions following 10-fold dilution. The behavior of investigated values of activity and concentration also differs in both groups of preparations: while concentration for the preparations not containing polymeric compounds showed on average a 10-fold change following dilution, the 10-fold dilution of preparations containing polymeric compounds resulted in an 18-to-20-fold change of these values.

These trends are not observed for mouthwash containing AmF (Lacalut Sensitive®) as a source of active fluorine, even though it contains polymeric compounds (polyethylene glycol) in its formulation; and the mode of change of both activity and concentration following dilution corresponds to that of mouthwashes that do not contain polymeric compounds. It is noteworthy that after each measurement of AmF mouthwash the electrode continued retaining the potential when being immersed in distilled water and even required additional washing or mechanical cleaning of membrane surface. Therefore, one could presume that high free fluoride to total fluoride ratio (94%) could be due to adsorption of AmF on the surface of the electrode membrane.

The determinable concentrations are almost identical for the Colgate Total Plax® mouthwashes, which only vary by type of coloring agent. However, for the Colgate Total Plax (Classic Mint®) mouthwash, the decrease in the activity of fluoride ions can be explained by the difference in the type of colorant added at approximately the same concentration of total fluoride. Therefore, it is reasonable to assume that the coloring agent added to Colgate Total Plax (Classic Mint®) mouthwash could bind free fluorides.

The analysis of the results, obtained for different series of the same mouthwash, revealed differences in the content of active fluorine for the Oral B® rinses and almost identical values for the Reach Fresh mint® solutions. The comparative analysis of test solutions containing NaF or AmF as a source of fluoride ions showed that there is no significant difference between the measured values of active fluorine and those calculated theoretically, in both procedures. Thus, both organic and inorganic sources of fluoride have similar potential against tooth decay, which can be confirmed by clinical data [29].

A particularly low activity of fluoride ions in comparison with the declared one was observed for the Lacalut Fresh® solution, in which AlF_3_ served as the source of fluoride ions. The results of determination of fluoride concentration by standard method showed an abnormal increase in the activity of fluoride ions following the initial dilution with TISAB and the standard behavior following the secondary dilution. When a standard of NaF was added to the solution, a sharp reduction of the potential was observed instead of an increase. Thus, the standard procedure for this preparation turned out to be inapplicable due to a possible interaction between the components of buffer solution TISAB and the components of the test solution. Therefore, to determine the total concentration of fluoride ions, the modified method was selected. To understand the practically 700-fold fluoride ion activity decrease compared to declared values, additional studies were performed, in particular the pH determination of test solutions. The results showed that Lacalut Sensitive® and Lacalut Fresh® solutions have the lowest pH values (4.2 and 4.4, respectively). In the experiment, the pH values of these solutions were adjusted to a value of 7.00, with the electromotive force for the Lacalut Sensitive® solution changing insignificantly (within 2%), whereas for Lacalut Fresh® the change was over 16%. To elucidate the reasons for the observed behavior, we added the precise aliquot of NaF standard solution to the test sample. The addition of the known amount of NaF led to a 6.5-fold increase in the activity of fluoride ions, whereas their theoretically calculated activity should have increased 100-fold. This allows one to conclude that the obtained result is the consequence of fluoride ion binding by the solution components into a non-ionized species of the fluorine compounds (complexes or poorly dissociating dimers hydrogen fluoride H_2_F_2_) [30]. To evaluate the binding capacity of fluoride ions, the experiment was carried out according to the modified method by doubling the concentration of the standard. In this case, the known concentration of NaF was added to the Lacalut Fresh® solution containing the standard until the activity doubled. The result of the experiment showed that the addition of 75% of the required amount of NaF was followed by the doubling of fluoride ion activity, with the further addition of a minimal amount of fluoride ions, leading to a dramatic increase in the activity (300-fold!). This provides quantitative proof of depletion of fluoride ion binding capacity (i.e., the component binding fluoride ions has completely reacted). Interestingly, the pH value of the solution dramatically changed: from 4,2 to 6.5 (the pH value of the standard solution of NaF is 7.7). The results of the experiment may be attributable to possible generation of strong complexes due to fluoride ion binding by the components of the test solution, particularly by exafluoroaluminate (AlF_3)_ salts (K_inst_ [AlF_6_]^3−^ = 2.1·10^− 21^) [31], rather than to formation of non-ionized fluorine compounds due to acidic pH value (e.g., the pH shift of the Lacalut Sensitive® solution resulted in insignificant changes). Thus, a study of fluoride ion activity in the Lacalut Fresh® solution suggests that fluoride ions will not be uptaken by tooth tissue and may even be washed away from it; thus, this supplement will have no effect when applied according to the directions for use of the preparation (5–7 drops/100 ml water).

The present study has some limitations since it does not reproduce in vivo conditions, i.e. dilution by saliva and influence of saliva components on the release of fluoride ions. It is known that phosphatase, one of saliva components, hydrolyzes Na_2_PO_3_F, releasing fluoride ions [25, 32]. Thus, it may be necessary to add components reproducing artificial saliva during sample preparation in order to avoid underestimated results when measuring Na_2_PO_3_F mouthwashes. The method of potentiometry itself does not account for the influence of mouthwash components on the work of the electrode.

## Conclusions

Mouthwashes are oral hygiene products aimed at enhancing the remineralization process and reducing demineralization due to the presence of fluoride ions; however, mouthwashes should also contain an appropriate amount of free fluorides to provide bioavailability. Ten different mouthwashes analyzed in the present study they revealed various free fluoride:total fluoride ratios due to binding of fluoride ions by mouthwash components or ability of fluoride source itself to form its complexes. The lower concentration of free fluoride in comparison with total one could lead to a decrease in the caries-preventive effect. However, the methods to quantify free fluoride have some disadvantages since they do not reflect in vivo conditions and, therefore, may result in distorted or underestimated values of fluoride content. Thus, there is a need to develop a method for evaluation of free fluorides in mouthwashes for proper updating of national and international guidelines.

## Data Availability

The data from this study are available upon request, which must be approved by all the authors.

## Abbreviations

Al^3+^: Aluminium ions
AlF_3_: Aluminum fluoride
AmF: Amine fluoride
Ca_10_(PO_4_)_6_(OH)_2_: Hydroxyapatite
Ca_10_(PO_4_)_6_(OH)F: Fluorohydroxyapatite
CaF_2_: Calcium fluoride
H_2_F_2_: Hydrogen fluoride
Na_2_PO_3_F: Sodium monofluorophosphate
Na_3_PO_4_: Trisodium phosphate
NaF: Sodium fluoride
NaOH: Sodium hydroxide
SnF_2_: Stannous fluoride
TISAB: Total ionic strength adjustment buffer

## References

[1] Treerutkuarkul A, Gruber K (2015). Prevention is better than treatment. Bull World Health Organ..

[2] Walsh T, Worthington HV, Glenny AM, Appelbe P, Marinho VC, Shi X. Fluoride toothpastes of different concentrations for preventing dental caries in children and adolescents. Cochrane Database Syst Rev. 2010;(1):CD007868. 10.1002/14651858.CD007868.pub2 20091655

[3] Rasines G (2010). Fluoride toothpaste prevents caries in children and adolescents at fluoride concentrations of 1000 ppm and above. Evid Based Dent..

[4] Marinho VC, Chong LY, Worthington HV, Walsh T (2016). Fluoride mouthrinses for preventing dental caries in children and adolescents. Cochrane Database Syst Rev.

[5] Marinho VC, Worthington HV, Walsh T, Chong LY. Fluoride gels for preventing dental caries in children and adolescents. Cochrane Database Syst Rev. 2015;(6):CD002280. 10.1002/14651858.CD002280.pub2 PMC7138249 26075879

[6] Twetman S, Keller MK (2016). Fluoride Rinses, Gels and Foams: An Update of Controlled Clinical Trials. Caries Res..

[7] Marinho VC, Worthington HV, Walsh T, Clarkson JE. Fluoride varnishes for preventing dental caries in children and adolescents. Cochrane Database Syst Rev. 2013;(7):CD002279. 10.1002/14651858.CD002279.pub2 PMC10758998 23846772

[8] Weyant RJ, Tracy SL, Anselmo TT, Beltran-Aguilar ED, Donly KJ, Frese WA (2013). Topical fluoride for caries prevention: executive summary of the updated clinical recommendations and supporting systematic review. J Am Dent Assoc.

[9] Sun Y, Li X, Deng Y, Sun JN, Tao D, Chen H (2014). Mode of action studies on the formation of enamel minerals from a novel toothpaste containing calcium silicate and sodium phosphate salts. J Dent..

[10] Zaze AC, Dias AP, Sassaki KT, Delbem AC (2014). The effects of low-fluoride toothpaste supplemented with calcium glycerophosphate on enamel demineralization. Clin Oral Investig..

[11] Takatsuka T, Tanaka K, Iijima Y (2005). Inhibition of dentine demineralization by zinc oxide: in vitro and in situ studies. Dent Mater..

[12] Blom T, Slot DE, Quirynen M, Van der Weijden GA (2012). The effect of mouthrinses on oral malodor: a systematic review. Int J Dent Hyg..

[13] Arweiler NB, Henning G, Reich E, Netuschil L (2002). Effect of an amine-fluoride-triclosan mouthrinse on plaque regrowth and biofilm vitality. J Clin Periodontol..

[14] Pizzo G, La Cara M, Licata ME, Pizzo I, D’Angelo M (2008). The effects of an essential oil and an amine fluoride/stannous fluoride mouthrinse on supragingival plaque regrowth. J Periodontol..

[15] Altenburger MJ, Schirrmeister JF, Wrbas KT, Hellwig E (2007). Remineralization of artificial interproximal carious lesions using a fluoride mouthrinse. Am J Dent..

[16] Laheij AM, van Strijp AJ, van Loveren C (2010). In situ remineralisation of enamel and dentin after the use of an amine fluoride mouthrinse in addition to twice daily brushings with amine fluoride toothpaste. Caries Res..

[17] Boyle P, Koechlin A, Autier P (2014). Mouthwash use and the prevention of plaque, gingivitis and caries. Oral Dis..

[18] Rugg-Gunn A, Banoczy J (2013). Fluoride toothpastes and fluoride mouthrinses for home use. Acta Med Acad.

[19] Faller RV, Eversole SL, Saunders-Burkhardt K (2014). Protective benefits of a stabilised stannous-containing fluoride dentifrice against erosive acid damage. Int Dent J..

[20] Garcia LSG, Delbem ACB, Pessan JP, Dos Passos Silva M, Neto FNS, Gorup LF, et al. Anticaries effect of toothpaste with nano-sized sodium hexametaphosphate. Clin Oral Investig. 2019;23(9):3535-42. 10.1007/s00784-018-2773-7 30539289

[21] Emerenciano NG, Botazzo Delbem AC, Pessan JP, Nunes GP, Souza Neto FN, de Camargo ER (2018). In situ effect of fluoride toothpaste supplemented with nano-sized sodium trimetaphosphate on enamel demineralization prevention and biofilm composition. Arch Oral Biol..

[22] Cheng LL (2017). Limited evidence suggests fluoride mouthrinse may reduce dental caries in children and adolescents. J Am Dent Assoc..

[23] Rosin-Grget K, Peros K, Sutej I, Basic K (2013). The cariostatic mechanisms of fluoride. Acta Med Acad..

[24] Zero DT (2006). Dentifrices, mouthwashes, and remineralization/caries arrestment strategies. BMC Oral Health..

[25] van Loveren C, Moorer WR, Buijs MJ, van Palenstein Helderman WH (2005). Total and free fluoride in toothpastes from some non-established market economy countries. Caries Res..

[26] Benzian H, Holmgren C, Buijs M, van Loveren C, van der Weijden F, van Palenstein Helderman W (2012). Total and free available fluoride in toothpastes in Brunei, Cambodia, Laos, the Netherlands and Suriname. Int Dent J..

[27] Carey CM, Holahan EC, Schmuck BD (2014). Analysis of 1-Minute Potentially Available Fluoride from Dentifrice. J Res Natl Inst Stand Technol..

[28] Martinez-Mier EA, Tenuta LMA, Carey CM, Cury JA, van Loveren C, Ekstrand KR (2019). European Organization for Caries Research Workshop: Methodology for Determination of Potentially Available Fluoride in Toothpastes. Caries Res..

[29] Ringelberg ML, Webster DB, Dixon DO, LeZotte DC (1979). The caries-preventive effect of amine fluorides and inorganic fluorides in a mouthrinse or dentifrice after 30 months of use. J Am Dent Assoc..

[30] Frant MS, Ross JW (1966). Electrode for sensing fluoride ion activity in solution. Science..

[31] Cardiano P, Cigala RM, Crea F, Giacobello F, Giuffre O, Irto A (2017). Sequestration of Aluminium (III) by different natural and synthetic organic and inorganic ligands in aqueous solution. Chemosphere..

[32] Duckworth RM, Knoop DT, Stephen KW (1991). Effect of mouthrinsing after toothbrushing with a fluoride dentifrice on human salivary fluoride levels. Caries Res..

